# Maternal Synbiotic Supplementation with *B. breve* M-16V and scGOS/lcFOS Shape Offspring Immune Development and Gut Microbiota at the End of Suckling

**DOI:** 10.3390/nu16121890

**Published:** 2024-06-15

**Authors:** Laura Sáez-Fuertes, Garyfallia Kapravelou, Blanca Grases-Pintó, Manuel Bernabeu, Karen Knipping, Johan Garssen, Raphaëlle Bourdet-Sicard, Margarida Castell, María Carmen Collado, Francisco José Pérez-Cano, María José Rodríguez-Lagunas

**Affiliations:** 1 Physiology Section, Department of Biochemistry and Physiology, Faculty of Pharmacy and Food Science, University of Barcelona (UB), 08028 Barcelona, Spain; laurasaezfuertes@ub.edu (L.S.-F.); gkapravelou@ub.edu (G.K.); blancagrases@ub.edu (B.G.-P.); margaridacastell@ub.edu (M.C.); mjrodriguez@ub.edu (M.J.R.-L.); 2 Nutrition and Food Safety Research Institute (INSA-UB), 08921 Santa Coloma de Gramenet, Spain; 3 Institute of Agrochemisty and Food Technology-National Research Council (IATA-CSIC), 46980 Valencia, Spain; mbernabeu@iata.csic.es (M.B.); mcolam@iata.csic.es (M.C.C.); 4 Danone Research & Innovation, 3584 Utrecht, The Netherlands; karen.knipping@ausnutria.nl (K.K.); johan.garssen@danone.com (J.G.); 5 Division of Pharmacology, Faculty of Science, Institute for Pharmaceutical Sciences, 3584 Utrecht, The Netherlands; 6 Danone Global Research & Innovation Center, 91 190 Gif, France; raphaelle.bourdet-sicard@danone.com; 7 Center for Biomedical Research Network for the Physiopathology of Obesity and Nutrition (CIBEROBN), Instituto de Salud Carlos III, 28029 Madrid, Spain

**Keywords:** breastfeeding, *Bifidobacterirum breve* M-16V, scGOS/lcFOS, immune system

## Abstract

Immune system development during gestation and suckling is significantly modulated by maternal environmental and dietary factors. Breastfeeding is widely recognized as the optimal source of nutrition for infant growth and immune maturation, and its composition can be modulated by the maternal diet. In the present work, we investigated whether oral supplementation with *Bifidobacterium breve M-16V* and short-chain galacto-oligosaccharide (scGOS) and long-chain fructo-oligosaccharide (lcFOS) to rat dams during gestation and lactation has an impact on the immune system and microbiota composition of the offspring at day 21 of life. On that day, blood, adipose tissue, small intestine (SI), mesenteric lymph nodes (MLN), salivary gland (SG), cecum, and spleen were collected. Synbiotic supplementation did not affect the overall body or organ growth of the pups. The gene expression of *Tlr9*, *Muc2*, *IgA*, and *Blimp1* were upregulated in the SI, and the increase in IgA gene expression was further confirmed at the protein level in the gut wash. Synbiotic supplementation also positively impacted the microbiota composition in both the small and large intestines, resulting in higher proportions of *Bifidobacterium* genus, among others. In addition, there was an increase in butanoic, isobutanoic, and acetic acid concentrations in the cecum but a reduction in the small intestine. At the systemic level, synbiotic supplementation resulted in higher levels of immunoglobulin IgG2c in plasma, SG, and MLN, but it did not modify the main lymphocyte subsets in the spleen and MLN. Overall, synbiotic maternal supplementation is able to positively influence the immune system development and microbiota of the suckling offspring, particularly at the gastrointestinal level.

## 1. Introduction

In humans, the newborn starts its development in the placenta, although it remains immature at birth. During pregnancy, maternal health status and environment directly influence fetus development [1], and the placenta serves as a communication bridge between the mother and the fetus by supplying the necessary nutrients [2]. Breastfeeding has been widely recognized as the most suitable nutrition for newborns The World Health Organization (WHO) suggests that newborns should be exclusively fed breast milk (BM) until 6 months of age [3]. During breastfeeding, an active communication is established between mother and infant through BM [4]. In addition, BM not only provides nourishment for the infant but also plays a role in the maturation of the infant’s immune system, contributing to “early life programming” [5]. BM is rich in bioactive components, including metabolites, vitamins, oligosaccharides, immunoglobulins (Igs), microbes, and microbial products, which are part of the passive immunity transfer to the newborn [6].

BM is a dynamic, complex fluid, and its composition changes depending on the lactation stage, infant’s needs, maternal diet, and environmental factors [7]. Over the last decade, research has proven that maternal nutrition modulates BM composition [8,9,10]. These approaches are aligned with the hypothesis that an intervention in the maternal diet is a potent alternative to support neonatal development through BM modulation [11]. In this sense, maternal diet supplemented with either probiotics, prebiotics, or synbiotics has been performed to study their impact on infant development [12].

Probiotics, prebiotics, and synbiotics have gained popularity in recent years. Probiotics are “live microorganisms which when administered in adequate amounts confer a health benefit on the host” [13]. Prebiotics are “a substrate that is selectively utilized by host microorganisms conferring a health benefit” [14]. Synbiotics are “a mixture comprising live microorganisms and substrate(s) selectively utilized by host microorganisms that confers a health benefit on the host” [15]. All of them contribute to maintaining and improving gastrointestinal and systemic health, and all are naturally present in BM [16].

The mechanism of action of pro-, pre-, and synbiotics first targets the gastrointestinal tract. To highlight some aspects, probiotics interact with the immune system by producing short chain fatty acids (SFCA) and inhibiting pathogen colonization [13]. Prebiotics work as immune modulators by promoting the proliferation of beneficial and probiotic bacteria and improving the gastrointestinal transit [14]; synbiotics combine both strategies to promote gastrointestinal health and gut barrier function [15].

Studies have shown that, during pregnancy and lactation, maternal gut microbiota may reach the mammary gland through the enteromammary pathway and modulate BM composition [17]. This mechanism of communication between the mother and the infant contributes to infant gut colonization and immune system maturation. Different studies have demonstrated the ability of probiotics to reach the mammary gland, influencing the infant’s fecal microbiota composition [18,19,20]. However, these studies have focused their research on the microbiota transference from the mother to the infant without evaluating the impact on the global infant immune system.

Suckling rats are still developing their immature immune system and become a good model to evaluate the impact of the administration of a bioactive component [21], as performed in previous studies, either directly to the pups [22,23] or through maternal intervention [20]. It is hypothesized that maternal intervention with the synbiotic will lead to an earlier acquisition of an immune pattern in line with more adult animals.

For this reason, the objective of the present study was to evaluate the impact of maternal supplementation during gestation and lactation with *Bifidobacterium breve* M-16V (10^9^ CFU) and short-chain galacto-oligosaccharide (scGOS) and long-chain fructo-oligosaccharide (lcFOS) at a ratio of 9:1 on the infant immune system at the end of suckling.

## 2. Materials and Methods

### 2.1. Animals and Experimental Design

Seven-week-old Lewis rats (16 females and 8 males) were obtained from Janvier Labs in La Plaine Saint Denis Cedex (France). Upon arrival, the rats had a one-week acclimatization period. After this period, the female rats were placed into the males’ cages for one week for mating. Then, the females were separated and placed into individual cages.

The female rats were then divided into two experimental groups: reference (REF) and synbiotic (SYN). Animals from the different groups were supplemented with a synbiotic (SYN, n = 8) or vehicle (REF, n = 8) during gestation (21 days) and lactation (21 days). The pups were allowed to be born naturally, and the day of birth was designated as day 1 of life for the pups. Litters were unified up to 10 pups per litter. Pups were divided to evaluate the impact at different ages: 4 pups/litter were euthanized on day 8 of life, 3 pups/litter and dams were euthanized on day 21 of life, and the remaining 3 pups/litter were euthanized on day 28 of life. Here, the results are focused on the pups euthanized on day 21 of life. Throughout the study, the rats had unrestricted access to a commercial diet formulated according to the American Institute of Nutrition 93G formulation [24]. They were also provided with water *ad libitum*, and the pups had free access to their mother’s nipples for nursing.

The animal room conditions (temperature and humidity) were controlled. The room followed a 12 h light–12 h dark cycle within a biosafety chamber at the Campus Diagonal Animal Facility of the Faculty of Pharmacy and Food Science at the University of Barcelona (UB). The experimental procedures conducted in this study were carried out with the necessary ethical approvals. The research received its approval from the Ethics Committee for Animal Experimentation (CEEA) of the University of Barcelona (Ref. 240/19) and from the Catalan Government (Ref. 10933). The procedure included the criteria used for including and excluding animals in the experiment. The sample size required was calculated by the Appraising Project Office’s program from the Universidad Miguel Hernández de Elche (Alicante). The minimal number of animals to provide statistically significant differences among groups, using the IgG plasma concentration as a variable and assuming that there was no dropout rate and type I error of 0.05 (two-sided), and that they proceed at least from more than 3 dams per group, as in previous studies, due to the high differences among litters.

The nutritional intervention started on the first day of gestation (G1) and continued until the end of the study, which was day 21 of the pups’ life (d21). The SYN group dams were orally administered 1 mL of a synbiotic mix daily during the gestation period and 1.5 mL during the lactation period, while the REF dams received an equivalent volume of a saline solution under similar conditions. Briefly, the synbiotic suspension was constituted by a mix of *Bifidobacterium breve* M-16V (10^9^ CFU/mL) with scGOS/lcFOS (9:1 proportion, 7.6 mg/mL and 0.08 mg/mL, respectively). The dose was calculated on the basis of a daily food intake of 40 g diet of a 2% prebiotic mixture. All supplements were kindly provided by Danone Research & Innovation (Utrecht, The Netherlands).

### 2.2. Sample Collection and Processing

The body weight of the animals was monitored daily, and at the end of the study the length of each animal was also measured. To assess the animals’ development and overall body composition the body mass index (BMI) ((*weight*/*length*^2^ (g/cm^2^)) and the Lee index (*weight*^0.33^/*length*) × 1000 (g^0.33^/cm) were calculated. In the middle of lactation (day 12 to day 14), feces were collected and frozen for probiotic tracking. Pups were anesthetized on day 21 to evaluate the impact of the maternal nutritional intervention during the breastfeeding period.

At the weaning day (day 21), pups were anesthetized with ketamine (90 mg/kg; Merial Laboratories S.A., Lyon, France) and xylazine (10 mg/kg; Bayer A.G., Leverkusen, Germany). Blood samples were obtained by cardiac exsanguination and analyzed in an automated haematologic analyzer (Spincell, MonLab Laboratories, Barcelona, Spain) or centrifuged to obtain plasma. Also, intestinal samples, adipose tissues (AT) (epididymal, parametric, dorsal, retroperitoneal, inguinal for white adipose tissue (WAT) and brown adipose tissue (BAT)), cecal content (CC), salivary gland (SG), mesenteric lymph nodes (MLN), and spleen were collected and immediately processed or stored at −20 °C or −80 °C for future analysis.

Adipose tissues (AT), both BAT and WAT (epidydimal or parametric, for males or females, respectively were chosen as representative of WAT), and small intestine (SI) were collected for histomorphometry. The central section of the SI was collected for gene expression analysis, embedding it in RNAlater (Ambion, Life Technology, Madrid, Spain), kept at 4 °C for 24 h, and stored at −20 °C later. The rest of the proximal part of the SI was opened lengthwise and cut into 0.5 cm pieces and incubated with PBS in a shaker (37 °C for 10 min) to obtain the gut wash (GW) for Ig quantification, and the content of the distal part of the intestine (IC) was collected for microbiota analysis.

### 2.3. Immunoglobulin Quantification

Plasma and homogenized MLN and SG were processed for ProcartaPlex™ Multiplex immunoassay (eBioscience, San Diego, CA, USA). Total IgA, IgM, IgG, and IgG isotypes (IgG1, IgG2a, IgG2b, IgG2c) were quantified following the manufacturer’s instructions as previously described [25] using a MAGPIX^®^ analyzer (Luminex Corporation, Austin, TX, USA) at the Cytometry Service of the Scientific and Technological Centers of the UB (CCiT-UB). The relative abundance of IgG subtypes was calculated with respect to the total IgG levels. IgG2b and IgG2c proportions allow one to assess the Th1 response, whereas IgG1 and IgG2a proportions allow one to obtain the Th2 response in rats [26].

Secretory (s) IgA in CC homogenates and GW samples was assessed by sandwich ELISA technique (Bethyl, Laboratories Inc., Montgomery, TX, USA). Also, IgM was quantified in GW by following the previously described protocol [27].

### 2.4. Tissue Histology

The central sections of the SI and the AT, selected for histomorphometry, were treated by immersing them in 4% buffered formaldehyde solution for 24 h at room temperature. Afterwards, the samples were rinsed in a phosphate-buffered solution (PBS) until dehydration in consequent graded ethanol solutions (70%, 90%, and 100%). After the dehydration and permeation steps in xylene, the samples were embedded in melted paraffin. Paraffin sections (5 µm) were stained using hematoxylin-eosin (HE). Olympus BX41 and Olympus XC50 cameras (Olympus, Barcelona, Spain) were used to examine the samples. For each sample of white adipose tissue (WAT) (20×), brown adipose tissue (BAT) (40×), and intestine (10×), representative photos were taken. All histology samples were analyzed using Image J (Image Processing and Analysis in Java, National Institute of Mental Health, Bethesda, MD, USA).

### 2.5. Gene Expression Analysis

RNAlater-stored SI samples were thawed for tissue homogenization using lysing matrix tubes and a FastPrep-24 instrument (MP biomedicals, Illkirch, France), as previously described [28]. RNA extraction was performed using the RNeasy Mini Kit (Qiagen, Madrid, Spain), following the manufacturer’s instructions. RNA purity and concentration were determined with a NanoPhotometer (BioNova Scientific S.L., Fremont, CA, USA), and cDNA was obtained using TaqMan Reverse Transcripiton Reagents (Applied Biosystems, AB, Weiterstadt, Germany). Then, real-time (RT) PCR was performed with an ABI Prism 7900 HT quantitative RT-PCR system (AB). Toll-like receptors (TLRs), barrier, and immune-related genes listed in Supplementary Table S1 were used.

After normalizing the results with the −2ΔΔCt method [29], data were showed as a percentage of expression in each experimental group with respect to the mean value obtained for the REF group, which was set at 100%.

Additionally, the identification of *B. breve* M-16V was conducted in the feces from day 12 to day 14 and CC at the end of suckling by extracting the DNA [26]. The TaqMan-based forward, reverse, and probe sequences were designed by Phavichitr et al. [30].

### 2.6. MLN and Spleen Lymphocytes Isolation

To isolate MLN and spleen lymphocytes, the tissues were passed through a sterile 40 μm mesh cell strainer (Thermo Fisher Scientific, Barcelona, Spain) using Roswell Park Memorial Institute (RPMI) 1640 medium (Sigma-Aldrich, Madrid, Spain), enriched with 10% fetal bovine serum (FBS, Sigma-Aldrich), 100 IU/mL streptomycin-penicillin (Sigma-Aldrich), 2 mM L-glutamine (Sigma-Aldrich), and 0.05 mM 2-β-mercaptoethanol (Merck Millipore, Darmstadt, Germany). The cell suspension was centrifuged at 538× *g* for 10 min at 4 °C. The resulting pellet was then resuspended in the RPMI-enriched medium. For splenic cells, an additional step was required to eliminate erythrocytes by osmotic lysis. Conditions were immediately restored by adding PBS to avoid lymphocyte death [31]. The number and viability of the cells were assessed using the Countess™ Automated Cell Counter (Invitrogen™, Thermo Fisher Scientific).

### 2.7. Cell Subset Staining and Flow Cytometry Analysis

MLN and spleen phenotypic populations were characterized by flow cytometry analysis using fluorescent mouse anti-rat monoclonal antibodies (mAbs) conjugated to different fluorochromes: fluorescein isothiocyanate (FITC), phycoerythrin (PE), peridinin chlorophyll protein (PerCP), allophycocyanin (APC), brilliant violet 421 (BV421), and phycoerythrin-Cyanine 7 (PE-Cγ7). All the mAbs were acquired from BD Biosciences, Serotec, and Caltag, respectively: anti-TCR (R73), anti-CD103 (OX-62), anti-NK (10/78), anti-CD62L (OX-85), anti-CD8 (OX-8), anti-CD4 (OX-35), anti-CD45RA (OX-33), and anti-TCR (V65). The staining technique was performed following the protocol previously described by Torres-Castro et al. [32]. Data were obtained with a Gallios^TM^ Cytometer (Beckman Coulter, Miami, FL, USA) in the CCiT-UB and analyzed by Flowjo v10 software (Tree Star, Inc., Ashland, OR, USA).

### 2.8. Cecal Bacteria and Ig-Coated Bacterial Analysis

Homogenized cecal samples were processed by flow cytometry to characterize the proportion of cecal bacteria and Ig-coated bacteria (Ig-CB), as in previous studies [33]. Data were collected with a Cytek Aurora (Cytek Biosciences, Inc., Fremont, CA, USA) in the CCTi-UB and analyzed using FlowJo v.10 software.

### 2.9. Short Chain Fatty Acids (SCFAs) Microbial Metabolite Profile

SCFA determination was performed using gas chromatography–mass spectrometry (GC-MS), as described previously by Eberhart et al. [34]. Samples with the addition of the internal standard solution (3-Methylvaleric acid) were centrifuged (1800× *g*, 2 min, 4 °C). The supernatant was filtered/sterilized (0.22 μm, Sarstedt SA, Nümbrecht, Germany) and then injected into the Agilent GC 7890B–5977B GC-MS with a multipurpose sampler (Gerstel MPS, Mülheim, Germany). The Agilent DB-FATWAX, 30 m × 0.25 mm × 0.25 μm GC column was used in split mode (20:1). The oven temperature program was 100 °C for 3 min, ramped to 100 °C at a rate of 5 °C/min, then to 150 °C for 1 min, then ramped to 200 °C at a rate of 20 °C/min, and finally held at 200 °C for 5 min. Helium was the carrier gas, and it was used at a flow rate of 1 mL/min, with an inlet temperature of 250 °C. The volume of injection was 2 μL. For the quantification of the SCFAs, standards curves for acetate, butyrate, and propionate were used.

### 2.10. Cecal Microbiota Profiling by 16S rRNA Amplicon Sequencing

Total DNA was isolated from the small intestine and CC (100–200 mg) using an automated assisted method based on magnetic beads (Maxwell^®^ RSC Instrument coupled with Maxwell RSC Pure Food GMO and authentication kit, Promega, Spain), following the manufacturer’s instructions with previous treatments to improve the DNA extraction. In brief, samples were treated with lysozyme and mutanolysin (20 mg/mL and 5 U/mL, respectively) for 60 min at 37 °C. After a preliminary step of cell disruption with 3 μm-diameter glass beads for 1 min at 6 m/s by a bead beater FastPrep 24-5 G Homogenizer (MP Biomedicals), the DNA obtained was purified using a DNA Purificaton Kit (Macherey-Nagel, Duren, Germany), and DNA concentration was measured using a Qubit^®^ 2.0 Fluorometer (Life Technology, Carlsbad, CA, USA). Microbial profiling was assessed by targeting the amplicon V3-V4 variable region of the 16S rRNA gene. Libraries were prepared following the 16S rDNA gene Metagenomic Sequencing Library Preparation Illumina protocol (Cod. 15044223 Rev. A). The libraries were then sequenced using a 2 × 300 bp paired-end run on a MiSeq-Illumina platform (FISABIO sequencing service, Valencia, Spain). Negative and positive mock community (Zymobiomics) communities were also included. Raw reads were then processed with the integrated dada2 method for denoising, amplicon sequence variance (ASV) clustering, and chimeral removal. Resulted ASV were then taxonomically assigned using Silva v.138.

### 2.11. Statistical Analysis

The statistical analysis utilized SPSS Statistics 22.0 software (SPSS Inc., Chicago, IL, USA). Normality and variance homogeneity of the data were evaluated using the Shapiro–Wilk and Levene tests, respectively. When the data followed normal and homogeneous distributions, a one-way ANOVA was conducted for analysis. In cases where the data did not follow normal and equal distributions, the Kruskal–Wallis test was employed to identify significant differences among groups (*p* < 0.05). Variable correlations were explored using the Spearman correlation coefficient. Non-metric multidimensional scaling (NMDS) was executed in R studio, employing the ‘vegan’ package [35], to identify clusters of sample similarities based on immune factor composition. The ‘envfit’ function was used to assess the association of factors with the ordination of samples in the NMDS plot. Statistical significance was established when the *p*-value < 0.05.

For microbiota composition analysis, no rarefaction was performed and samples with less than 4500 reads were removed, and data was normalized using centered-log-ratio (CLR). Beta diversity analysis was based on the Bray–Curtis distances matrix, and permutational analysis of variance (PERMANOVA) was performed. The alpha-diversity indexes Chao1 and Shannon were also calculated, and differences by group were assessed by Mann–Whitney and/or Kruskal–Wallis non-parametric test. Besides this, the Kruskall-Wallis test on the CLR-normalized data were also assessed with Benjamini–Hochberg false discovery rate (FDR) correction. Negative binomial regression, as implemented by the DESeq2 tool, was used for differential abundance analysis to estimate the fold-change of genus taxa [36]. Plots were generated using MicrobeAnalyst platform v.2 [37].

## 3. Results

### 3.1. Growth and Morphometry

**Figure 1:**
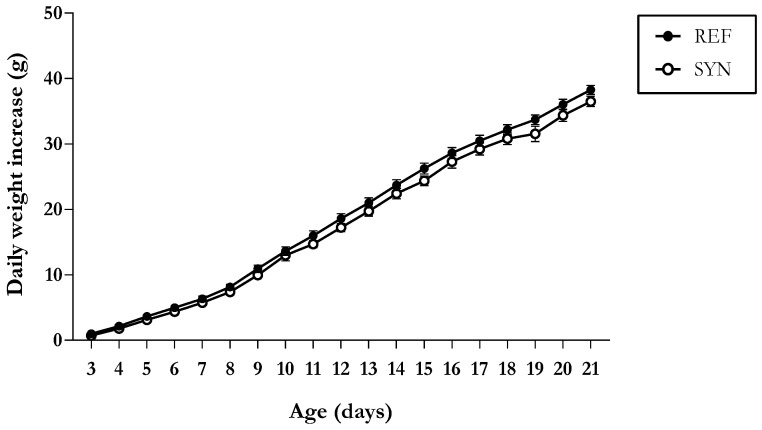
Daily weight increase with respect to day 2 of neonatal rats during the suckling period. Results are expressed as mean ± standard error of the mean (S.E.M.) (n = 11–16).

Body weight evolution was assessed throughout the study (Figure 1), and both groups revealed a similar growth pattern, without differences between sexes.

At the end of suckling, pups were also measured to calculate different growth-associated parameters such as the body/tail length ratio, the BMI, and the Lee Index (Supplementary Table S2). The SYN group did not exhibit any significant changes in the measured parameters compared to the REF group. After exsanguination, organ weights were recorded, and maternal synbiotic supplementation did not affect the overall values. However, in the SYN group there was a decrease in the relative weight of the cecum and an increase in both relative weight and length of the SI (Supplementary Table S2).

### 3.2. Intestinal Morphology

**Figure 2:**
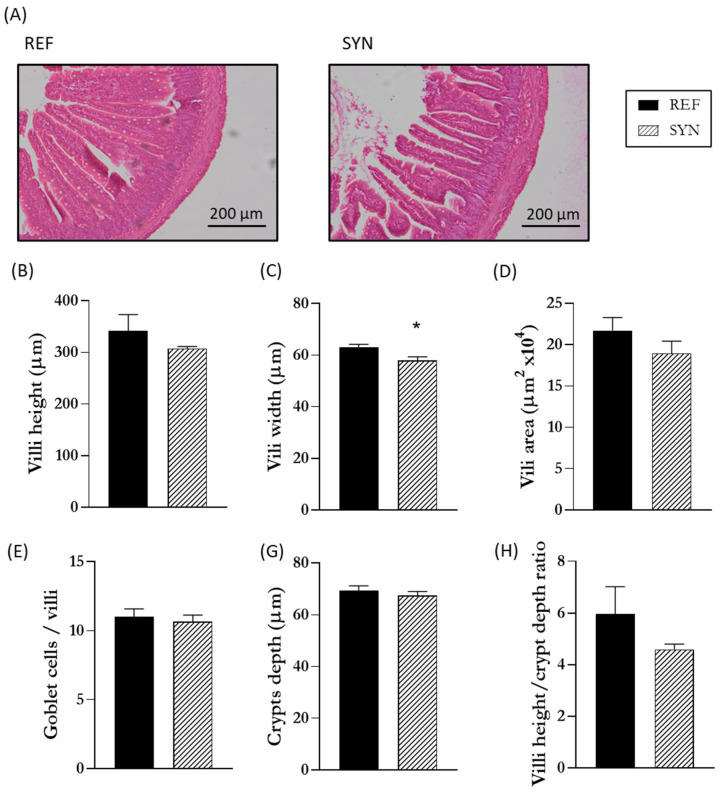
Effect of maternal synbiotic supplementation on the pups’ intestinal morphology. (**A**) Representative images of the small intestine stained with hematoxylin and eosin, 10×. (**B**) Height, (**C**) width, (**D**) area of the intestinal villi, (**E**) ratio of goblet cells/villi, (**F**) crypts depth, and (**G**) ratio of the villi height/crypt depth. Results are expressed as mean ± S.E.M. Statistical differences: * *p* < 0.05 vs. REF (n = 11–16).

To follow the trophic effect in the SI of 21-day-old rats after maternal supplementation during gestation and lactation, a histomorphometric analysis of the SI was conducted (Figure 2). In the SYN group, no significant changes were observed in the villi height and area, crypts depth, or goblet cells abundance. However, a reduction in villi width was observed at the end of the suckling period.

### 3.3. Intestinal Gene Expression

**Figure 3:**
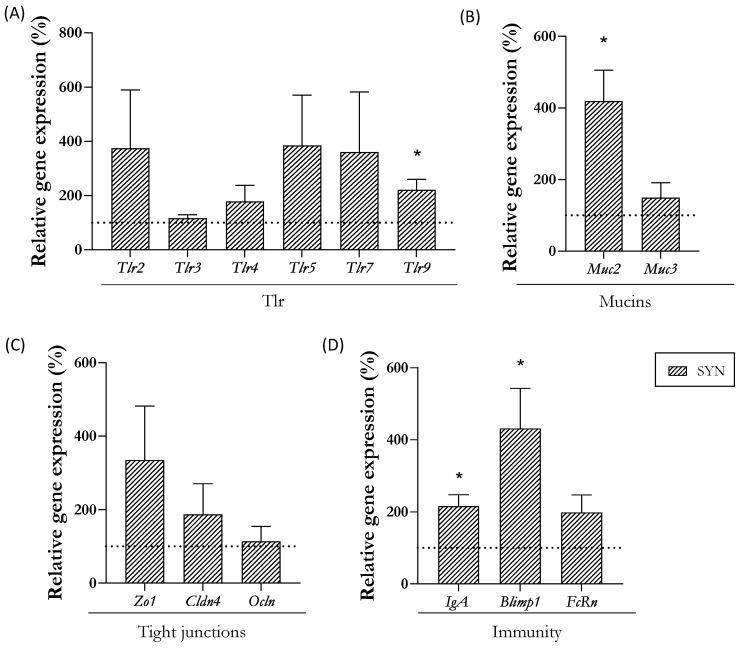
Impact of maternal synbiotic supplementation in the small intestine. Relative gene expression analysis in the small intestine of (**A**) Toll-like receptors (TLR), (**B**) mucins, (**C**) tight-junction (TJ) proteins, and (**D**) immunological proteins. Relative gene expression was calculated with respect to REF, which corresponded to 100% of transcription (represented with a horizontal dotted line). Statistical differences: * *p* < 0.05 vs. REF (n = 11–16). *TLR*, Toll-like receptor; *Muc*, mucin; *Zo*-*1*, zonula occludens-1; *Cldn4*, claudin 4; *Ocln*, occludin; *IgA*, immunoglobulin A; *Blimp1*, B lymphocyte-induced maturation protein-1; *FcRn*, neonatal Fc receptor.

The expression levels of genes involved in defense, mucosal barrier, and immunity were also assessed at day 21 (Figure 3). The supplemented group displayed an increase in the gene expression of the *Tlr9*, whereas the *Tlr2*, *3*, *4*, *5*, and *7* remained unaffected (Figure 3A). The analysis of mucins revealed that *Muc2* gene expression was upregulated in the SYN group (Figure 3B). The proteins associated with epithelial barrier functions, such as tight-junction (TJ) proteins, were not modulated by maternal supplementation (Figure 3C). Among the genes involved in the immunological intestinal status (*IgA* and *Blimp1*)*,* its gene expression was increased at the end of the study (Figure 3D).

### 3.4. Small and Large Intestinal Immunity Ig Profile

**Figure 4:**
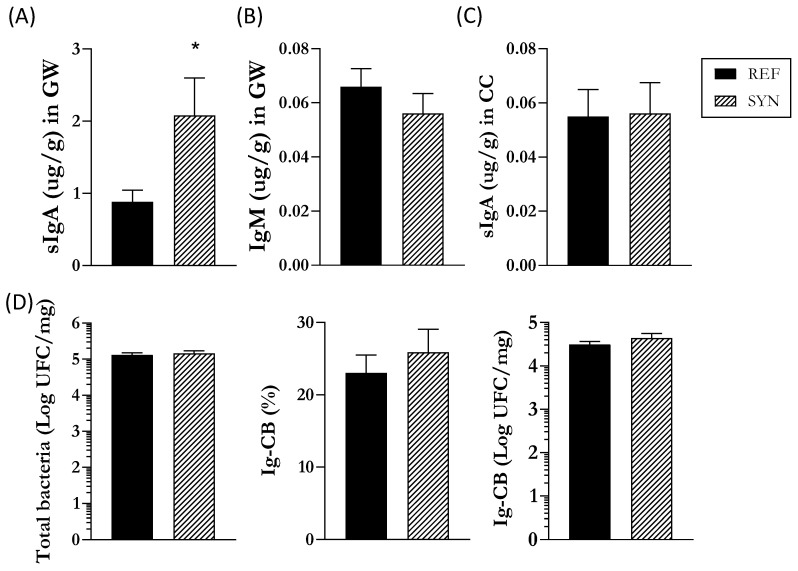
Impact of maternal supplementation at the end of the lactation on the gastrointestinal tract of pups. Quantification of (**A**) IgA and (**B**) IgM in the gut wash and (**C**) IgA in the cecum. (**D**) Analysis of cecal bacteria composition counts of total bacteria, proportion of Ig-CB, total Ig-CB. Data are expressed as mean ± S.E.M. Statistical differences: * *p* < 0.05 vs. REF (n = 11–16).

First, the analysis of the SI was completed by studying the Ig composition of the GW (Figure 4). The SYN group exhibited higher sIgA levels in the intestine at the end of lactation than those in the REF group, whereas IgM remained unaffected (Figure 4A,B).

Second, analysis of the cecal composition was performed (Figure 4). Differentially to that found in the SI, the total levels of cecal sIgA were not modulated with maternal nutritional intervention (Figure 4C). As cecal Igs bind to cecal bacteria to promote their neutralization and elimination [38], the number of total bacteria and the proportion of Ig-CB in the CC were also evaluated. No changes were observed, either in the total bacteria or in the relative Ig-CB. However, a clear tendency toward an increase (*p* = 0.06) of the total Ig-CB was found in the SYN group (Figure 4D).

### 3.5. Microbial SCFAs Production

**Figure 5:**
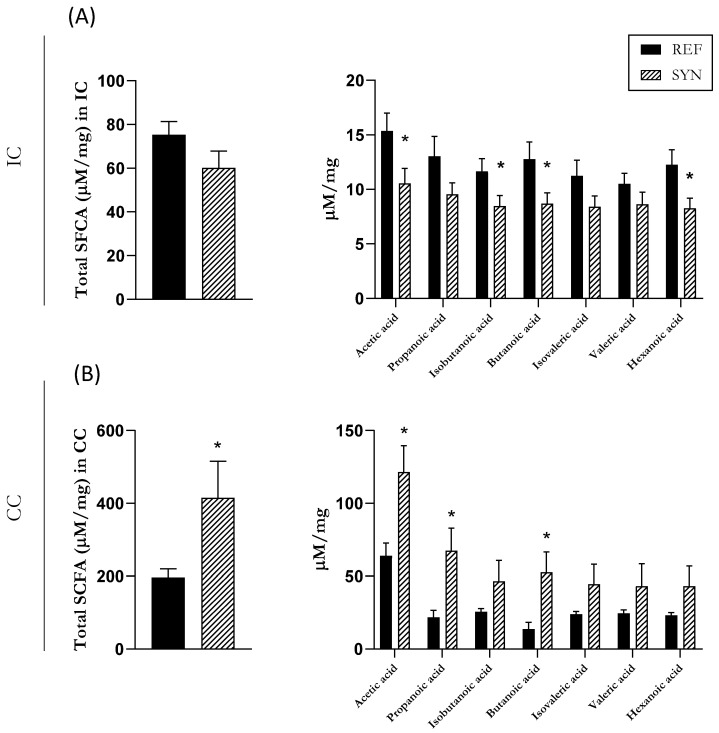
Short-chain fatty acid (SCFA) production in the pups’ rat gastrointestinal tract. (**A**) SCFAs in intestine content (IC) and (**B**) in cecal content (CC). Data are expressed as mean ± S.E.M. Statistical differences: * *p* < 0.05 vs. REF (n = 11–16).

The main mechanism of communication between the cecal microbiota and the immune system is through the production of SCFAs. Thus, the quantification of SCFA levels serves as an indicator of microbiota functionality [39]. Even though SCFAs predominate in the cecum, the intestinal levels were also analyzed (Figure 5). The composition of the IC was evaluated, and although no changes were observed in the total amount of SCFAs, a reduction in the acetic, isobutanoic, butanoic, and hexanoic acids was observed at the end of the study in the SYN group (Figure 5A). In contrast, in the cecum, the total production of the SCFAs increased at weaning in the SYN group, and this increase was mainly due to an increase in the acetic, propanoic, and butanoic acids (Figure 5B).

### 3.6. Small Intestinal and Cecal Microbiota

**Figure 6:**
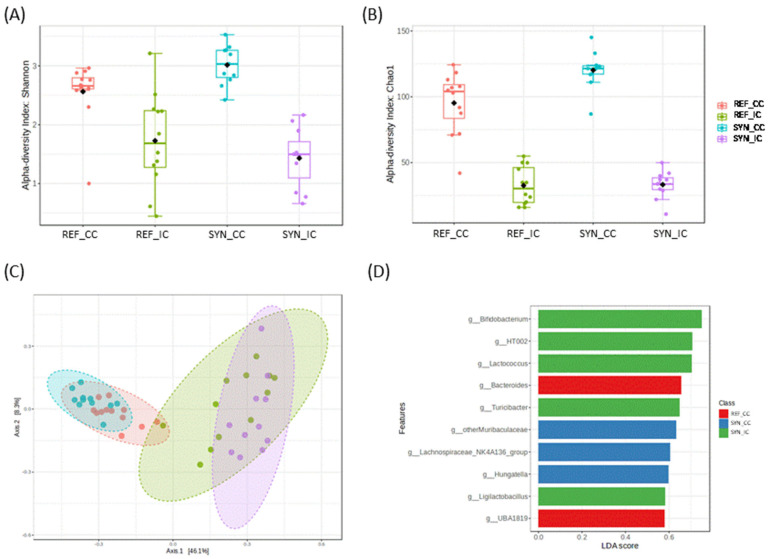
Alpha−diversity indexes (**A**) (Shannon index) and (**B**) richness (Chao1 index) for CC and IC. (**C**) Beta −diversity in cecal content (CC) and intestinal content (IC) microbiota depending on SYN intervention. (**D**) Linear discriminant analysis (LDA) effect size (LEfSe) plot of taxonomic genera identified in CC and CI. Statistical testing was performed by PERMANOVA using Bray–Curtis distances, and the Mann–Whitney test was used for alpha-diversity indexes. Black square in figures (**A**,**B**) indicate the mean value (n = 5–6).

**Figure 7:**
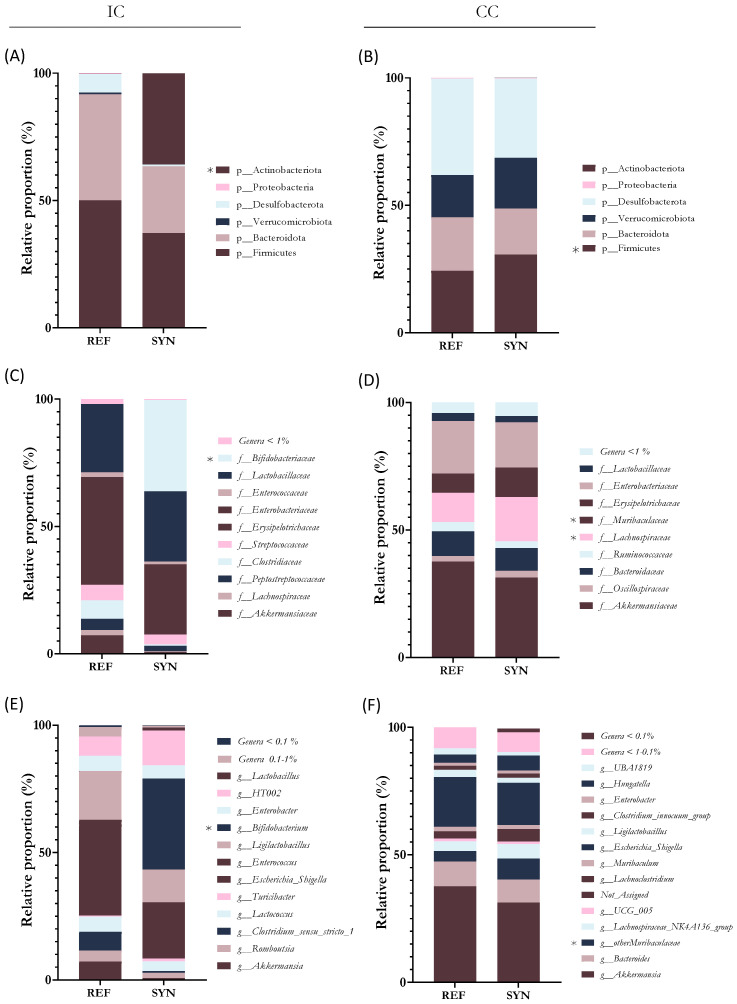
Microbiota analysis of the small intestinal content (IC) and cecum content (CC). Relative bacterial proportions at phylum level in (**A**) IC and (**B**) CC. Relative proportions of IC microbiota at family level in (**C**) IC and (**D**) CC. Relative proportions of IC microbiota at genus level in (**E**) IC and (**F**) CC. Results are expressed as relative proportions for each population. Statistical differences: * *p* < 0.05 vs. REF(n = 5–6).

The analysis of the microbiota composition was performed in terms of phylum, family, and genus (Figure 6 and Figure 7).

Alpha diversity and beta-diversity analysis showed significant differences, depending on the sample type (intestine vs. cecum) and depending on the intervention (REF vs. SYN). In detail, regarding the alpha-diversity metrics, cecum showed significantly higher microbial diversity (Shannon index) and richness (Chao1 index) compared to the intestine. SYN increased significantly the microbial richness (Chao1) and diversity (Shannon) in CC (*p* = 0.0053 and *p* = 0.015, respectively); however, no effects were found in the small intestine (CI, *p* = 0.829 and *p* = 0.413, respectively) (Figure 6A,B).

Beta-diversity showed two general distinct microbial clusters, depending on sample-type CC vs. IC, accounting for 54.4% of the variation (PERMANOVA test F-value: 14.0; R-squared: 0.50; *p* = 0.001) (Figure 6C). In addition, when groups were compared considering the intervention, we observed a significant impact of the SYN supplementation on the cecum (PERMANOVA test F-value: 4.1159; R-squared: 0.164; *p* = 0.001) and on the intestine (PERMANOVA F-value: 2.69; R-squared: 0.113; *p* = 0.013) (Supplementary Figure S1). DESEq tests showed the differential presence of specific microbial genera. An enrichment in *Bifidobacterium* genus (FDR *p* < 0.001), *Faecalibaculum* (FDR *p* < 0.001), *Lactobacillus* (FDR *p* = 0.027), and *Turicibacter* genus (FDR *p* = 0.036) was observed after the SYN intervention in CI. In CC, the SYN intervention also increased the *Prevotellaceae*_UCG_001 (FDR *p* < 0.001). LEfSe test also demonstrated the role of these microbial genera, depending on SYN intervention (Figure 6D).

Regarding bacterial proportions, in the small intestine, the most abundant phylum was Firmicutes in the REF group. However, in the SYN group, the supplementation induced an increase in the Actinobacteria phylum (mainly by *Bifidobacterium* genus) (Figure 7A). Evaluating the abundance of *Bifidobacteriaceae* family proportion it increased by up to a 35% in the SYN (*p* = 0.261) group compared to the REF (Figure 7C). The analysis of the genus proportion confirmed the family results, showing higher proportions of *Bifidobacterium* genus at weaning in the SYN group (Figure 7E).

In the cecum, Firmicutes, and Desulfobacterota were the most abundant phyla, and the Firmicutes increased significantly after the SYN group (Figure 7B). The family analysis revealed that maternal supplementation modified the composition of the offspring CC; *Bifidobacteria* were only present in the SYN group and *Lanchospiracea* and *Muribacteriaceae* proportions were increased (Figure 7D). The genus analysis revealed that *Bifidobacterium* and *Faecalibaculum* were only detected in the SYN group, and *Muribaculaceae* was increased after maternal nutritional intervention (Figure 7F). Additionally, *B. breve* M-16V was detected by qPCR in the CC of the SYN group at ~3.5 × 10^9^ UFC/mg at the end of suckling. However, at the middle of lactation, the probiotic was detected in only 30% of the analyzed fecal samples and at very low levels (>10^7^ UFC/mg).

### 3.7. Lymphocyte Populations

The synbiotic supplementation to dams affects the infant’s gastrointestinal tract during lactation, potentially influencing lymphocytic populations at the intestinal level, but also spreading to the systemic compartment.

**Figure 8:**
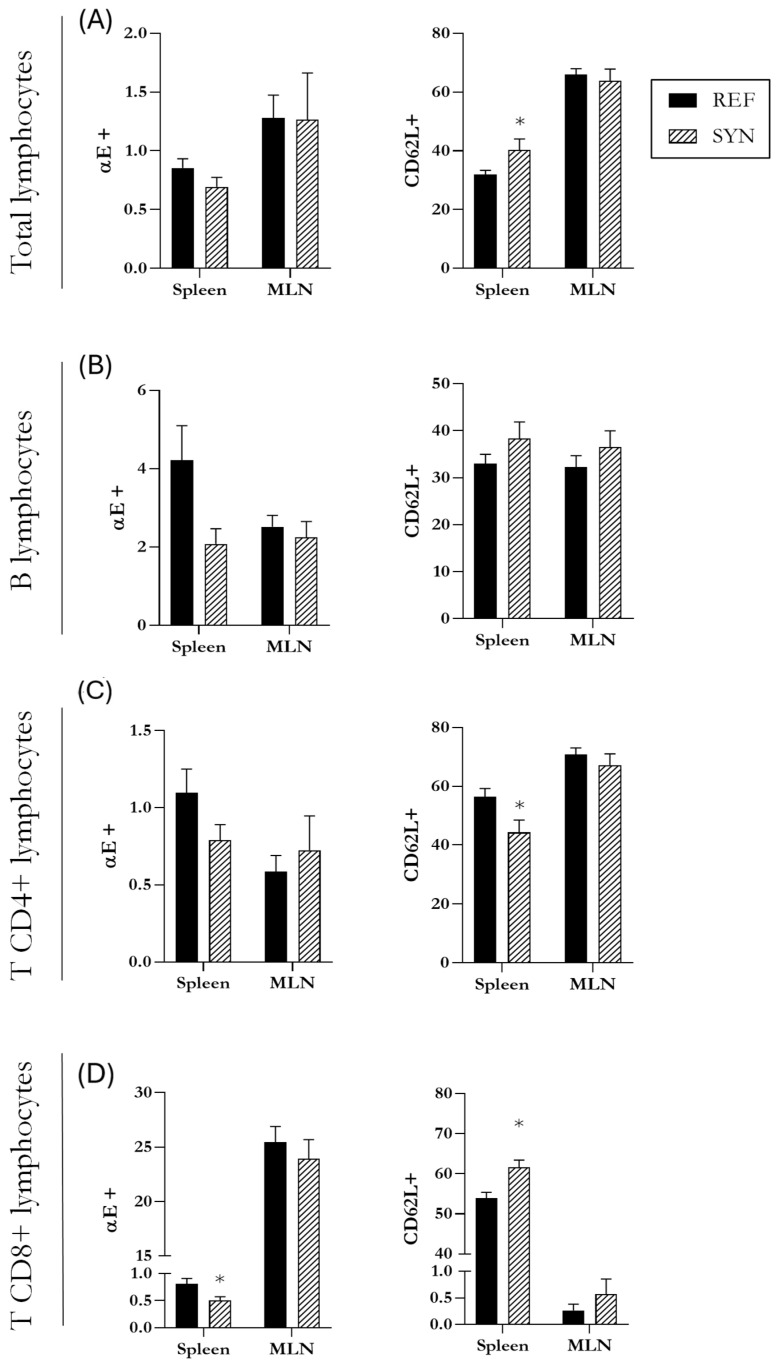
Analysis of the expression of migration markers CD62L and αE in different lymphocytes subsets in the spleen and mesenteric lymph nodes (MLN). Evaluation of the expression of CD62L and αE in the total lymphocytes (**A**), in B cell lymphocytes (**B**), in T CD4+ lymphocytes (**C**), and T CD8+ lymphocytes (**D**). Data are expressed as mean ± S.E.M. Statistical differences: * *p* < 0.05 vs. REF (n = 11–16).

At the intestinal level, the MLN cells/populations were studied; detailed data are shown in Table S3. The proportion of B cells accounted for ~20%, whereas the T cells were ~70%. None of them were affected at the weaning day due to synbiotic intervention. The T cells subsets, T CD4+ and T CD8+, were approximately ~47% and ~17%, respectively. The NK cell proportion was higher in the SYN group at the end of suckling with respect to the REF group, whereas NKT were not affected. Additionally, two migration markers (CD62L+ and αE+) were assessed in the total lymphocyte subsets, in B cells, in T CD4+ cells, and in T CD8+ cells (Figure 8). The results indicated that they were not altered with maternal synbiotic supplementation.

To enrich the analysis of the lymphocyte populations, the evaluation of the lymphocytes was also performed in the spleen, a secondary lymphoid tissue representative of systemic immune system (Table S3). B cells and T cells in this tissue equally constituted approximately 30% of the lymphocytes, and none of these populations were affected by maternal supplementation. When all lymphocytes are analyzed, including both B and T populations, the proportion of cells expressing f CD62L+ was higher in the SYN than in the REF group in the spleen (Figure 8A). Regarding B cells, neither activation levels (CD25+) nor the expression of migration markers (CD62L+ and αE+) were altered in the SYN group (Figure 8B). In terms of T cells, the relative proportion of T CD4+ or T CD8+ was not affected. However, the expression of the CD62L selectin was lower in the T CD4+ population and higher in the T CD8+ from the SYN animals than in the REF ones (Figure 8C,D). Additionally, the proportion of αE+ in T CD8+ from SYN animals was lower than that in the REF group.The NK and NKT population percentages were not modified either.

In addition, analysis of the blood cell composition was performed (Supplementary Table S4), and no significant changes were observed in leukocyte counts, erythrocytes, or platelets.

### 3.8. Immunoglobulin Profile in Different Tissues

**Figure 9:**
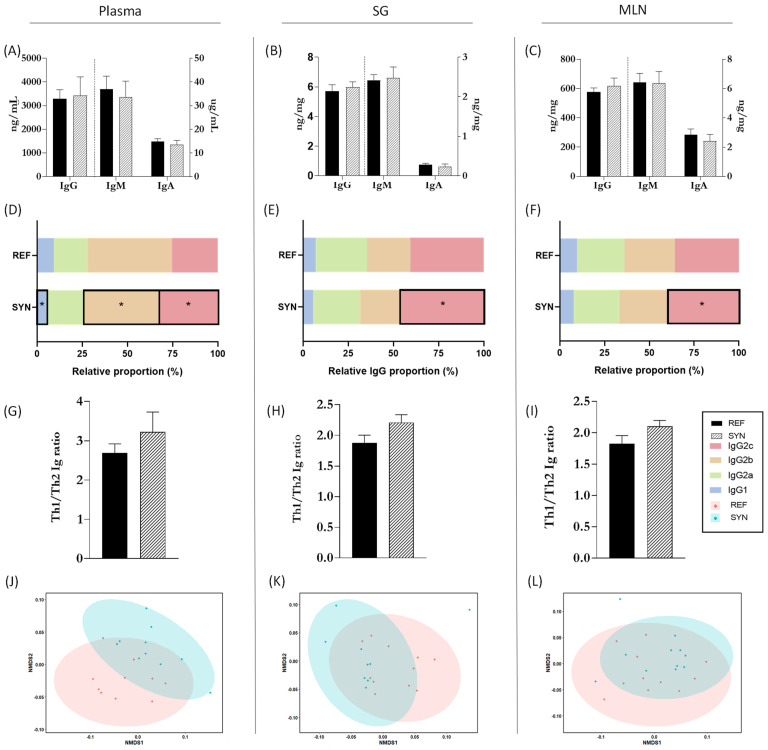
Immunoglobulin quantification in pups at day 21 of life in different compartments. Total Igs levels (IgA, IgM, and IgG) in (**A**) plasma, (**B**) SG, and (**C**) MLN. Relative proportion of IgG subtypes in (**D**) plasma, (**E**) SG, and (**F**) MLN. Analysis of the Th1/Th2 ratio in plasma (**G**), SG (**H**), and MLN (**I**). Analysis of non-parametric multidimensional scaling (NMDS) for the Ig profiles based on the Bray–Curtis distance in (**J**) plasma, (**K**) SG, and (**L**) MLN. Data (**A**–**I**) are expressed as mean ± S.E.M. The vertical dotted line (**A**–**C**) splits the graph in two parts, on the left units and scale bar are different from those on the right. Each point in NMDS (**J**−**L**) represents an animal (**C**) by ANOSIM test. Statistical differences: * *p* < 0.05 vs. REF (n = 11–16).

Igs levels, as valuable markers of immunological status [40], were assessed at the end of suckling in the systemic and mucosal compartments (Figure 9). Total levels of IgM, IgA, and IgG in plasma, SG, and MLN were unaffected by maternal supplementation (Figure 9A–C). However, there were variations in the relative proportions of IgG subtypes. Notably, IgG2c showed a higher proportion in SYN compared to REF animals in all three compartments (Figure 9D–F). The Th1/Th2 ratio was evaluated using the levels of the IgG2b and IgG2c (representing Th1) and IgG1 and IgG2a (representing Th2). Overall, the synbiotic nutritional intervention did not affect the Th1/Th2 balance in the offspring (Figure 9G–I). After assessing Ig levels in each compartment, NMDS graphs were generated to identify distinct clusters based on global Ig profiles (Figure 9J–L). In this study, maternal synbiotic supplementation did not lead to the differentiation of clusters in any of the evaluated compartments.

### 3.9. Adipose Tissue (AT) Analysis

**Figure 10:**
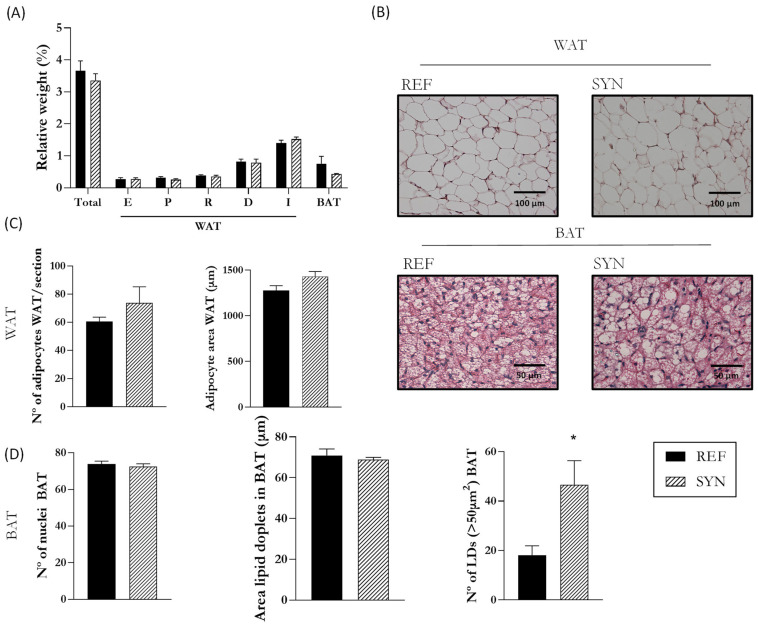
Influence of maternal supplementation on AT. (**A**) Relative weight of the adipose tissue from different locations. (**B**) Histological analysis of parametric WAT (P-WAT) and BAT in hematoxylin and eosin-stained sections. Images were captured at 20× and 40× magnification, respectively. (**C**) Analysis of WAT, epidydimal, or parametric, for males or females, respectively, were chosen as representative of the WAT: adipocyte area and number of adipocytes. (**D**) Analysis of BAT: number of nuclei, area of lipid droplets (LD), and number of LD (>50 µm^2^). Data are expressed as mean ± S.E.M. Statistical differences: * *p* < 0.05 vs. REF (n = 11–16). WAT, white adipose tissue; BAT, brown adipose tissue; E, epididymal; P, parametric; R, retroperitoneal; D, dorsal; I, Inguinal.

The impact of maternal supplementation was also evaluated on the AT of their descendants at the end of suckling (Figure 10). Nutritional intervention during gestation and lactation did not affect the relative AT weight of the pups (Figure 10A). Representative images of histologic sections of WAT and BAT are shown in Figure 10B. The quantification of the number of adipocytes and their area were not modified in the WAT tissue (Figure 10C). Likewise, the number of nuclei and the area of the lipid droplets in the BAT were not altered. However, large lipid droplets, considered as those larger than 50 µm^2^, were more abundant in the BAT of the SYN animals than in the REF ones (Figure 10D).

## 4. Discussion

Good nutritional habits during pregnancy and breastfeeding present an opportunity to improve the health of both mother and child [41,42,43,44]. Among the maternal environmental factors, diet before conception and during pregnancy and breastfeeding has been linked to the modulation of breast-milk composition, which in turn influences the immunological status of both the mother and the offspring [12,45,46]. Concretely, prebiotic supplementation during pregnancy and lactation has been targeted as a potential strategy to boost both maternal and offspring immune system and microbiota composition [20].

However, there is very little information available about the potential impact of maternal supplementation with probiotics, prebiotics, or their combination to mothers and their infants during gestation and lactation on overall infant development. Our results clearly indicate that a supplementation of maternal diet with *Bifidobacterium breve* M-16V and scGOS/lcFOS during gestation and lactation improves the mucosal immune system and microbiota composition of the neonate at the end of suckling. The observed effects may be due to the direct impact of these components on several maternal variables by strengthening the intestinal barrier, improving the microbial composition, and promoting an improved immune response. All these effects could be derived by indirect effects on the pups during gestation through placenta or breast milk (i.e., by higher metabolites or Ig transfer). This indirect beneficial effect on the offspring was previously confirmed when a probiotic was administered to the mothers [20].

To assess the influence of maternal synbiotic supplementation on offspring growth, we initially examined its effects on macroscopic and morphologic variables. As the daily growth pattern was similar between the groups, it can be suggested that maternal synbiotic supplementation did not affect the overall growth until weaning. In line with this, Rigo-Adrover et al. did not find any differences on the body weight evolution when the same synbiotic was administered directly to the pups during early life [23]. In the present study, the analysis of growth-related parameters and the relative organ weights confirmed that maternal synbiotic supplementation did not have an effect. However, a trophic effect in the SI by its increasing weight and length was observed, suggesting an increase in the intestinal surface, and favoring the absorption capacity of nutrients of the pups [47]. Similar results were found when the scGOS/lsFOS was administered to rotavirus-infected pups in early life [48].

After evaluating the macroscopic effects of SYN supplementation on the SI, we proceeded to analyze its impact at the microscopic level to validate the observed trophic effect. Our findings indicated that, while both the length and weight of the SI increased, most of the microscopic structures remained unaffected (including the villi height and area, the crypts depth, the villi height and the crypt depth ratio, and the amount of goblet cells). Only a slight reduction in villi width was observed at weaning. The SI acts as a barrier between the external environment and the host. Its primary function is nutrient absorption, whereas the villi and microvilli of the intestine optimize this absorption [49]. For this reason, an increase of the area of villi and microvilli structures is expected to enhance nutrient absorption. Supplementation with probiotics and prebiotics has been associated with positive effects on the homeostasis and integrity of the gastrointestinal tract. Many studies have analyzed the impact of biotics on the intestinal structures. Most of them were focused on the direct administration of pro-, pre-, or synbiotics [22,50,51]. However, few studies have focused on the effect of maternal supplementation on the offspring intestine. Wang et al. supplemented pregnant pigs during gestation with a synbiotic mix (*Lactiplantibacillus plantarum*, *Saccharomyces cerevisiae*, and xylo-oligosaccharides), and they observed that maternal supplementation influenced the intestinal structures of the offspring by increasing the villi height [52]. Our results are not in accordance with these data; however, it needs to be taken under consideration the host difference and that the effect of the synbiotics are dose- and strain-dependent.

In addition to the analysis of morphological structures, we evaluated the effect of maternal synbiotic supplementation on the gene expression levels of relevant intestinal markers, including TLRs, mucins, TJ proteins, and maturation genes. The synbiotic supplementation induced higher *Tlr9*, *Muc2*, *IgA*, and *Blimp1* gene expression. *Tlr9* plays a pivotal role in innate immunity, safeguarding internal homeostasis against danger signals [53]. *Muc2* is the main constituent of the small intestine’s mucus layer, offering protection to the intestinal tract against self-digestion and facilitating nutrient retention for efficient nutritional uptake [54,55]. Previous studies have demonstrated that synbiotic pups’ supplementation with a *Bifidobacterium* strain and 2′-Fucosyllactose also increased *Muc2* gene expression in the intestine [56]. In healthy conditions, an increase of sIgA is considered as a positive outcome as sIgA interacts with pathogenic organisms, preventing their penetration into the intestinal barrier [57]. The enhancement of this sIgA agrees with the one described by Wang et al., who demonstrated that a maternal synbiotic supplementation in pigs during gestation and lactation also increased the intestinal sIgA of the offspring [52]. Finally, the *Blimp1* maturation marker is expressed during fetal and neonatal periods, and its expression decreases at weaning [58]. However, our results did not reveal such a decrease at the end of suckling. In general, our results suggest that maternal synbiotic supplementation induced a positive effect on the SI by increasing the expression of *Tlr9*, *Muc2*, and *IgA*. These genes will contribute to maintaining intestinal homeostasis and avoiding pathogen colonization. Nonetheless, further studies are needed to evaluate the impact of maternal nutrition intervention on intestinal maturation and reveal the mechanism that leads to this increase in the supplemented mothers.

To corroborate the intestinal gene expression, sIgA and IgM protein levels were assessed. According to the RT-PCR results, sIgA was increased in the SYN pups, suggesting higher pathogen neutralization capacity [57]. Intestinal IgM interacts with different antigens by opsonizing (coating) them, favoring their destruction. Interestingly, we did not detect any significant changes in intestinal IgM levels following synbiotic supplementation. These results remained consistent, even after the SYN mix was administered directly to pups, as described in previous studies [23].

The importance of cecal immune composition has been rising in the last decade due to the established link between microbiota and the immune system. Within the cecum, sIgA plays a crucial role by coating cecal bacteria and participating in their neutralization to facilitate their subsequent elimination [38]. Maternal synbiotic supplementation did not exert any effect on the total sIgA, nor in the Ig-CB at the end of suckling in this compartment. Ongoing studies on the impact of such a mixture on the dams allow one to observe that this change observed in the pups was also found at the maternal level and then, somehow, also transferred to the pups.

Besides the immune observations at intestinal level, the microbiota composition and activity were also modulated by maternal supplementation. Despite observing no changes in the total amount of bacteria in the cecum, we examined the proportion of the different microbiota proportions in the intestine and cecum. Notably, the SYN group exhibited higher proportions of *Bifidobacterium* in the intestinal compartment, with the detection of *Bifidobacterium* and *Faecalibaculum* exclusively in the cecum. *Bifidobacterium* spp., particularly *B. breve*, *B. bifidum*, and *B. longum* subsp. *infantis*, are predominant, and these are known for their positive impact on infant development, including immune system maturation [59,60,61]. *Faecalibaculum* spp. are novel species of potential beneficial microorganism productors of SCFAs. Additionally, *Facecalibaculum* contributes to reducing colon inflammation by stimulating Treg cells [62]. These results suggest that synbiotic maternal supplementation during gestation and lactation boosts the colonization of the offspring gut during suckling.

Thus, although the supplemented probiotic strain *B. breve* M-16Vused was not detected in the supplemented BM from supplemented dams nor in all the feces at the middle lactation, it was detected in the CC of the SYN pups at the end of suckling. It is noteworthy that some days before day 21 of life, the pups had already initiated chewing. Consequently, the SYN pups might chew the maternal feces, acquiring the administered probiotic, explaining why the strain is detected in pups at day 21 but not in pups at the middle of lactation. It is important to highlight that *B. breve* M-16V was not detected in the REF group in the feces nor in the CC. Similar data were found when a probiotic (*L. fermentum* CECT5716) was supplemented to the dams; only some samples of the offspring were strain positive at the middle lactation [20]. These data cannot confirm that maternal synbiotic supplementation may be transferred to the offspring through their mother’s milk, as it was not detected after analysis, but it cannot be discarded either and requires further studies focused on the transmission pathway.

SCFAs are recognized as mediators of communication between the intestinal microbiota and the immune system. The major SCFAs include formic, acetic, propionic, and butyric acids [63]. Our findings indicated that maternal synbiotic supplementation induced an increase in the total SCFAs in the offspring cecum. This rise was linked to higher levels of acetic, propanoic, and butanoic acids. In the SI, some of the SCFAs were lower, although the total levels were not affected. However, it must be considered that the production of SCFAs is mainly linked to the cecum. The production of each specific acid is linked to different bacterial groups [39]. Butyric acid is known for its antioxidant and anti-inflammatory properties, contributing to the maintenance of digestive and immune homeostasis [64]. Additionally, an increase in butyric acid has been linked with an increase of intestinal *Muc2* gene expression via the selective acetylation/methylation of histones. This observation agrees with our results, where maternal synbiotic supplementation induced an increase of butyric acid and the intestinal *Muc2* gene expression in the offspring [65]. Acetic acid production is linked to the protection of epithelial cells and the promotion of probiotic bacteria growth [66], and it is positively correlated with fecal sIgA [67]. Similar results were obtained in our study, where maternal synbiotic supplementation increased the acetic acid in the cecum and the intestinal IgA in the offspring during pregnancy. Propionic acid has been associated with anti-proliferative effects and improvements in lipid metabolism and serves as a precursor of gluconeogenesis in the liver [68,69,70]. Formic acid has been less studied, but it is known to be linked to methanogenesis and inflammation [71,72]. Numerous studies have consistently shown that probiotic supplementation provokes an increase in the production of SCFA [39,73,74,75]. Joining the microbiota and the SCFAs results in the IC, it is important to focus on the reduction of some of the SCFAs, and it can be associated with the reduced bacterial diversity in the IC. However, in the CC, the increase of cecal butyrate, acetate, and propionate can be linked to the increase of the Firmicutes phylum in the CC, as they are the main producers of these acids [76]. All these data support our hypothesis that maternal synbiotic supplementation during gestation and lactation positively impacts infant cecum microbiota and its SCFAs production due to the observed increase at the end of suckling. In the future, more research is needed to investigate whether higher levels of SCFAs derived from synbiotic supplementation are transferred to the fetus through the placenta.

Regarding immunity, the influence of maternal synbiotic supplementation extended beyond the biomolecular level, affecting various lymphocyte subset populations in both mucosal (MLN) and systemic (spleen) compartments. In general, maternal synbiotic supplementation did not exert great changes on these lymphocyte populations. It can be highlighted that, in the spleen, an increase in the migration marker CD62L was observed at weaning due to synbiotic supplementation. CD62L is a migration marker linked to cells that are ready to migrate to the intestinal compartment [77]. Little information is known about the impact of diet supplementation on lymphocyte subset populations. Only a few studies are available; in those cases, the supplementation was typically administered directly to the animals rather than being provided as maternal supplementation [78]. Thus, it is a new unexplored field that should be addressed in the future, as it can elegantly show the potential of the maternal diet on lymphocyte cell numbers and even activity.

To complete the analysis, the Ig profile of different samples was performed. The impact of pro-, pre-, and synbiotics on the Ig production has been documented [79]. Our results indicated that maternal synbiotic intervention, administered during both gestation and lactation, did not lead to significant modifications in the IgA, IgM, and IgG isotypes in offspring plasma, SG, or MLN. However, the IgG subtype proportions in pups were modulated after maternal nutritional intervention. Mainly, the IgG2c levels were increased in the plasma, SG, and MLN samples. While limited information is available regarding the role of IgG2c in rats and mice, its analog IgG3 has been associated with regulatory responses in the intestine and is involved in long-term immunity [80,81]. The observed rise in IgG2c levels is consistent with previous findings in which increased IgG2c levels were documented in both the milk and plasma of dams following the administration of the same SYN mix (unpublished results).

Finally, the impact of maternal synbiotic supplementation on the morphology of adipose tissue was performed. In recent years, there has been more and more research focused on the influence of maternal nutrition during pregnancy on the offsprings’ adipose tissue [82,83]. However, there is a lack of information on the influence of synbiotic supplementation during pregnancy and lactation. Our results suggest that maternal nutritional intervention did not modulate the adipose tissue metabolism; only a punctual rise in large-sized lipid droplets of the BAT was observed. Lukaszewski et al. found that the maternal dietary pattern influences adipose tissue programming in the offspring. However, they did not review the impact of synbiotic supplementations [84]. Therefore, further studies are needed.

Maternal supplementation with *Bifidobacterium breve* M-16V and scGOS/lcFOS on the offspring exerts positive effects; however, there are some limitations to this study. Specifically, there is a need to further determine the relative contribution of gestation and lactation periods to the immune maturation of the pups. Additionally, while the health benefits of the synbiotics are highly documented, future research should aim to elucidate whether *B. breve* M-16V or scGOS/lcFOS plays a more pivotal role in immune system modulation. Furthermore, the study did not explore all possible mechanisms underlying immune system modulation. In addition, although in vivo studies in rats have contributed to understanding the mechanisms in humans, in some cases, the results’ integration may be difficult. Finally, the study has focused on the physiological conditions of a healthy pregnancy and normal development of the offspring. However, with the effects found in the pups after the maternal supplementation established under the healthy conditions of the mother and the offspring, a future approach could involve investigating the synbiotic impact in pathological or infectious conditions in both the mother and the offspring.

## 5. Conclusions

Maternal synbiotic supplementation during both gestation and lactation exerts a positive influence on the infant’s immune system development. The main remarkable effects are observed within the gastrointestinal tract, in which maternal supplementation enhances the integrity and function of the intestinal barrier and microbiota composition. Thus, this strategy appears to be a useful tool to improve mucosal immunity and the intestinal barrier of the offspring. Nevertheless, further research is necessary to assess the long-term implications of maternal synbiotic supplementation during pregnancy and lactation on both mothers and offspring.

## Data Availability

The Illumina sequencing raw data were uploaded to the NCBI database.

## Supplementary Materials

The following supporting information can be downloaded at: https://www.mdpi.com/article/10.3390/nu16121890/s1, Figure S1: Analysis of non-parametric multidimensional scaling (NMDS) for the microbiota profiles based on the Bray-Curtis distance; Table S1: Description of the specific TaqMan primers AB; Table S2: Growth-associated parameters and organ’s size at the weaning day; Table S3: Effect of maternal synbiotic supplementation on the spleen and MLN lymphocyte population at weaning; Table S4: Haematological variables at day 21 of pups’ life.

## References

[1] Bibbò S., Ianiro G., Giorgio V., Scaldaferri F., Masucci L., Gasbarrini A., Cammarota G. (2016). The Role of Diet on Gut Microbiota Composition. Eur. Rev. Med. Pharmacol. Sci..

[2] Gude N.M., Roberts C.T., Kalionis B., King R.G. (2004). Growth and Function of the Normal Human Placenta. Thromb. Res..

[3] Breastfeeding. https://www.who.int/health-topics/breastfeeding#tab=tab_1.

[4] Vieira Borba V., Sharif K., Shoenfeld Y. (2018). Breastfeeding and Autoimmunity: Programing Health from the Beginning. Am. J. Reprod. Immunol..

[5] Björkstén B. (1999). The Intrauterine and Postnatal Environments. J. Allergy Clin. Immunol..

[6] Andreas N.J., Kampmann B., Mehring Le-Doare K. (2015). Human Breast Milk: A Review on Its Composition and Bioactivity. Early Hum. Dev..

[7] Lis J., Orczyk-Pawiłowicz M., Ka̧tnik-Prastowska I. (2013). [Proteins of Human Milk Involved in Immunological Processes]. Postepy Hig. Med. Dosw..

[8] Selma-Royo M., González S., Gueimonde M., Chang M., Fürst A., Martínez-Costa C., Bode L., Collado M.C. (2022). Maternal Diet Is Associated with Human Milk Oligosaccharide Profile. Mol. Nutr. Food Res..

[9] Innis S.M. (2014). Impact of Maternal Diet on Human Milk Composition and Neurological Development of Infants. Am. J. Clin. Nutr..

[10] Mohammad M.A., Sunehag A.L., Haymond M.W. (2009). Effect of Dietary Macronutrient Composition under Moderate Hypocaloric Intake on Maternal Adaptation during Lactation 1–5. Am. J. Clin. Nutr..

[11] Rio-Aige K., Azagra-Boronat I., Castell M., Selma-Royo M., Collado M.C., Rodríguez-Lagunas M.J., Pérez-Cano F.J. (2021). The Breast Milk Immunoglobulinome. Nutrients.

[12] Bravi F., Wiens F., Decarli A., Dal Pont A., Agostoni C., Ferraroni M. (2016). Impact of Maternal Nutrition on Breast-Milk Composition: A Systematic Review. Am. J. Clin. Nutr..

[13] Hill C., Guarner F., Reid G., Gibson G.R., Merenstein D.J., Pot B., Morelli L., Canani R.B., Flint H.J., Salminen S. (2014). Expert Consensus Document: The International Scientific Association for Probiotics and Prebiotics Consensus Statement on the Scope and Appropriate Use of the Term Probiotic. Nat. Rev. Gastroenterol. Hepatol..

[14] Gibson G.R., Hutkins R., Sanders M.E., Prescott S.L., Reimer R.A., Salminen S.J., Scott K., Stanton C., Swanson K.S., Cani P.D. (2017). Expert Consensus Document: The International Scientific Association for Probiotics and Prebiotics (ISAPP) Consensus Statement on the Definition and Scope of Prebiotics. Nat. Rev. Gastroenterol. Hepatol..

[15] Swanson K.S., Gibson G.R., Hutkins R., Reimer R.A., Reid G., Verbeke K., Scott K.P., Holscher H.D., Azad M.B., Delzenne N.M. (2020). The International Scientific Association for Probiotics and Prebiotics (ISAPP) Consensus Statement on the Definition and Scope of Synbiotics. Nat. Rev. Gastroenterol. Hepatol..

[16] Eriksen K.G., Christensen S.H., Lind M.V., Michaelsen K.F. (2018). Human Milk Composition and Infant Growth. Curr. Opin. Clin. Nutr. Metab. Care.

[17] Jost T., Lacroix C., Braegger C.P., Rochat F., Chassard C. (2014). Vertical Mother-Neonate Transfer of Maternal Gut Bacteria via Breastfeeding. Environ. Microbiol..

[18] Abrahamsson T.R., Sinkiewicz G., Jakobsson T., Fredrikson M., Björkstén B. (2009). Probiotic Lactobacilli in Breast Milk and Infant Stool in Relation to Oral Intake during the First Year of Life. J. Pediatr. Gastroenterol. Nutr..

[19] Martín V., Maldonado-Barragán A., Moles L., Rodriguez-Baños M., Campo R.D., Fernández L., Rodríguez J.M., Jiménez E. (2012). Sharing of Bacterial Strains Between Breast Milk and Infant Feces. J. Hum. Lact..

[20] Azagra-Boronat I., Tres A., Massot-Cladera M., Franch À., Castell M., Guardiola F., Pérez-Cano F.J., Rodríguez-Lagunas M.J. (2020). *Lactobacillus fermentum* CECT5716 Supplementation in Rats during Pregnancy and Lactation Affects Mammary Milk Composition. J. Dairy Sci..

[21] Pérez-Cano F.J., Franch Á., Castellote C., Castell M. (2012). The Suckling Rat as a Model for Immunonutrition Studies in Early Life. Clin. Dev. Immunol..

[22] Azagra-Boronat I., Massot-Cladera M., Mayneris-Perxachs J., Knipping K., Van’t Land B., Tims S., Stahl B., Garssen J., Franch À., Castell M. (2019). Immunomodulatory and Prebiotic Effects of 2′-Fucosyllactose in Suckling Rats. Front. Immunol..

[23] Rigo-Adrover M., Saldaña-Ruíz S., van Limpt K., Knipping K., Garssen J., Knol J., Franch A., Castell M., Pérez-Cano F.J. (2017). A Combination of ScGOS/LcFOS with *Bifidobacterium breve* M-16V Protects Suckling Rats from Rotavirus Gastroenteritis. Eur. J. Nutr..

[24] Reeves P.G., Nielsen F.H., Fahey G.C. (1993). AIN-93 Purified Diets for Laboratory Rodents: Final Report of the American Institute of Nutrition Ad Hoc Writing Committee on the Reformulation of the AIN-76A Rodent Diet. J. Nutr..

[25] Ruiz-Iglesias P., Massot-Cladera M., Rodríguez-Lagunas M.J., Franch À., Camps-Bossacoma M., Castell M., Pérez-Cano F.J. (2022). A Cocoa Diet Can Partially Attenuate the Alterations in Microbiota and Mucosal Immunity Induced by a Single Session of Intensive Exercise in Rats. Front. Nutr..

[26] Gracie J.A., Bradley J.A. (1996). Interleukin-12 Induces Interferon-Gamma-Dependent Switching of IgG Alloantibody Subclass. Eur. J. Immunol..

[27] Massot-Cladera M., Franch À., Castellote C., Castell M., Pérez-Cano F.J. (2013). Cocoa Flavonoid-Enriched Diet Modulates Systemic and Intestinal Immunoglobulin Synthesis in Adult Lewis Rats. Nutrients.

[28] Camps-Bossacoma M., Pérez-Cano F.J., Franch À., Untersmayr E., Castell M. (2017). Effect of a Cocoa Diet on the Small Intestine and Gut-Associated Lymphoid Tissue Composition in an Oral Sensitization Model in Rats. J. Nutr. Biochem..

[29] Pérez-Cano F.J., Ramírez-Santana C., Molero-Luís M., Castell M., Rivero M., Castellote C., Franch À. (2009). Mucosal IgA Increase in Rats by Continuous CLA Feeding during Suckling and Early Infancy. J. Lipid Res..

[30] Phavichitr N., Wang S., Chomto S., Tantibhaedhyangkul R., Kakourou A., Intarakhao S., Jongpiputvanich S., Roeselers G., Knol J. (2021). Impact of Synbiotics on Gut Microbiota during Early Life: A Randomized, Double-Blind Study. Sci. Rep..

[31] Estruel-Amades S., Ruiz-Iglesias P., Périz M., Franch À., Pérez-Cano F.J., Camps-Bossacoma M., Castell M. (2019). Changes in Lymphocyte Composition and Functionality After Intensive Training and Exhausting Exercise in Rats. Front. Physiol..

[32] Torres-Castro P., Grases-Pintó B., Abril-Gil M., Castell M., Rodríguez-Lagunas M.J., Pérez-Cano F.J., Franch À. (2020). Modulation of the Systemic Immune Response in Suckling Rats by Breast Milk TGF-Β2, EGF and FGF21 Supplementation. Nutrients.

[33] Pérez-Berezo T., Franch A., Ramos-Romero S., Castellote C., Pérez-Cano F.J., Castell M. (2011). Cocoa-Enriched Diets Modulate Intestinal and Systemic Humoral Immune Response in Young Adult Rats. Mol. Nutr. Food Res..

[34] Eberhart B.L., Wilson A.S., O’Keefe S.J.D., Ramaboli M.C., Nesengani L.T. (2021). A Simplified Method for the Quantitation of Short-Chain Fatty Acids in Human Stool. Anal Biochem..

[35] Dixon P. (2003). VEGAN, a Package of R Functions for Community Ecology. J. Veg. Sci..

[36] Love M.I., Huber W., Anders S. (2014). Moderated Estimation of Fold Change and Dispersion for RNA-Seq Data with DESeq2. Genome Biol..

[37] Lu Y., Zhou G., Ewald J., Pang Z., Shiri T., Xia J. (2023). MicrobiomeAnalyst 2.0: Comprehensive Statistical, Functional and Integrative Analysis of Microbiome Data. Nucleic Acids Res..

[38] Mantis N.J., Rol N., Corthésy B. (2011). Secretory IgA’s Complex Roles in Immunity and Mucosal Homeostasis in the Gut. Mucosal Immunol..

[39] Markowiak-Kopeć P., Śliżewska K. (2020). The Effect of Probiotics on the Production of Short-Chain Fatty Acids by Human Intestinal Microbiome. Nutrients.

[40] Alberts B., Johnson A., Lewis J., Raff M., Roberts K., Walter P. (2002). B Cells and Antibodies. Molecular Biology of the Cell.

[41] Amati F., Hassounah S., Swaka A. (2019). The Impact of Mediterranean Dietary Patterns during Pregnancy on Maternal and Offspring Health. Nutrients.

[42] Moon R.J., Citeroni N.L., Aihie R.R., Harvey N.C. (2023). Early Life Programming of Skeletal Health. Curr. Osteoporos. Rep..

[43] Moreno-Mendez E., Quintero-Fabian S., Fernandez-Mejia C., Lazo-De-La-Vega-Monroy M.L. (2020). Early-Life Programming of Adipose Tissue. Nutr. Res. Rev..

[44] Langley-Evans S.C. (2022). Early Life Programming of Health and Disease: The Long-term Consequences of Obesity in Pregnancy. J. Hum. Nutr. Diet..

[45] Ramakrishnan U., Grant F., Goldenberg T., Zongrone A., Martorell R. (2012). Effect of Women’s Nutrition before and during Early Pregnancy on Maternal and Infant Outcomes: A Systematic Review. Paediatr. Perinat. Epidemiol..

[46] García R.M.M., Ortega A.I.J., Peral-Suárez Á., Bermejo L.M., Rodríguez-Rodríguez E. (2021). [Importance of Nutrition during Pregnancy. Impact on the Composition of Breast Milk]. Nutr. Hosp..

[47] Okpe C.G., Abiaezute N.C., Adigwe A. (2016). Evaluation of the Morphological Adaptations of the Small Intestine of the African Pied Crow (Corvus Albus). J. Basic Appl. Zool..

[48] Azagra-Boronat I., Massot-Cladera M., Knipping K., Van’t Land B., Stahl B., Garssen J., José Rodríguez-Lagunas M., Franch À., Castell M., Pérez-Cano F.J. (2018). Supplementation With 2′-FL and ScGOS/LcFOS Ameliorates Rotavirus-Induced Diarrhea in Suckling Rats. Front. Cell Infect. Microbiol..

[49] Judkins T.C., Archer D.L., Kramer D.C., Solch R.J. (2020). Probiotics, Nutrition, and the Small Intestine. Curr. Gastroenterol. Rep..

[50] Morales-Ferré C., Azagra-Boronat I., Massot-Cladera M., Tims S., Knipping K., Garssen J., Knol J., Franch À., Castell M., Pérez-Cano F.J. (2022). Preventive Effect of a Postbiotic and Prebiotic Mixture in a Rat Model of Early Life Rotavirus Induced-Diarrhea. Nutrients.

[51] Yi H., Wang L., Xiong Y., Wen X., Wang Z., Yang X., Gao K., Jiang Z. (2018). Effects of Lactobacillus Reuteri LR1 on the Growth Performance, Intestinal Morphology, and Intestinal Barrier Function in Weaned Pigs. J. Anim. Sci..

[52] Wang K., Hu C., Tang W., Azad M.A.K., Zhu Q., He Q., Kong X. (2021). The Enhancement of Intestinal Immunity in Offspring Piglets by Maternal Probiotic or Synbiotic Supplementation Is Associated With the Alteration of Gut Microbiota. Front. Nutr..

[53] Saber M.M., Monir N., Awad A.S., Elsherbiny M.E., Zaki H.F. (2022). TLR9: A Friend or a Foe. Life Sci..

[54] Johansson M.E.V., Hansson G.C. (2016). The Mucins. Encycl. Immunobiol..

[55] Ermund A., Schütte A., Johansson M.E.V., Gustafsson J.K., Hansson G.C. (2013). Studies of Mucus in Mouse Stomach, Small Intestine, and Colon. I. Gastrointestinal Mucus Layers Have Different Properties Depending on Location as Well as over the Peyer’s Patches. Am. J. Physiol. Gastrointest. Liver Physiol..

[56] Luo Y., Zhang Y., Yang Y., Wu S., Zhao J., Li Y., Kang X., Li Z., Chen J., Shen X. (2023). Bifidobacterium Infantis and 2′-Fucosyllactose Supplementation in Early Life May Have Potential Long-Term Benefits on Gut Microbiota, Intestinal Development, and Immune Function in Mice. J. Dairy Sci..

[57] Pietrzak B., Tomela K., Olejnik-Schmidt A., Mackiewicz A., Schmidt M. (2020). Secretory IgA in Intestinal Mucosal Secretions as an Adaptive Barrier against Microbial Cells. Int. J. Mol. Sci..

[58] Arévalo Sureda E., Weström B., Pierzynowski S.G., Prykhodko O. (2016). Maturation of the Intestinal Epithelial Barrier in Neonatal Rats Coincides with Decreased FcRn Expression, Replacement of Vacuolated Enterocytes and Changed Blimp-1 Expression. PLoS ONE.

[59] Milani C., Hevia A., Foroni E., Duranti S., Turroni F., Lugli G.A., Sanchez B., Martín R., Gueimonde M., van Sinderen D. (2013). Assessing the Fecal Microbiota: An Optimized Ion Torrent 16S RRNA Gene-Based Analysis Protocol. PLoS ONE.

[60] Turroni F., Peano C., Pass D.A., Foroni E., Severgnini M., Claesson M.J., Kerr C., Hourihane J., Murray D., Fuligni F. (2012). Diversity of Bifidobacteria within the Infant Gut Microbiota. PLoS ONE.

[61] Hidalgo-Cantabrana C., Delgado S., Ruiz L., Ruas-Madiedo P., Sánchez B., Margolles A. (2017). Bifidobacteria and Their Health-Promoting Effects. Microbiol. Spectr..

[62] Hu Q., Yu L., Zhai Q., Zhao J., Tian F. (2023). Anti-Inflammatory, Barrier Maintenance, and Gut Microbiome Modulation Effects of Saccharomyces Cerevisiae QHNLD8L1 on DSS-Induced Ulcerative Colitis in Mice. Int. J. Mol. Sci..

[63] Morrison D.J., Preston T. (2016). Formation of Short Chain Fatty Acids by the Gut Microbiota and Their Impact on Human Metabolism. Gut Microbes.

[64] Vieira A., Ramirez Vinolo M. (2019). Regulation of Immune Cell Function by Short Chain Fatty Acids and Their Impact on Arthritis. Bioactive Food as Dietary Interventions forArthritis and Related Inflammatory Diseases.

[65] Burger-van Paassen N., Vincent A., Puiman P.J., van der Sluis M., Bouma J., Boehm G., van Goudoever J.B., Van Seuningen I., Renes I.B. (2009). The Regulation of Intestinal Mucin MUC2 Expression by Short-Chain Fatty Acids: Implications for Epithelial Protection. Biochem. J..

[66] Fukuda S., Toh H., Taylor T.D., Ohno H., Hattori M. (2012). Acetate-Producing Bifidobacteria Protect the Host from Enteropathogenic Infection via Carbohydrate Transporters. Gut Microbes.

[67] Blaak E.E., Canfora E.E., Theis S., Frost G., Groen A.K., Mithieux G., Nauta A., Scott K., Stahl B., van Harsselaar J. (2020). Short Chain Fatty Acids in Human Gut and Metabolic Health. Benef. Microbes.

[68] Sa A., Henningsson M., Margareta E., Nyman G.L., Björck I.M.E. (2001). Content of Short-Chain Fatty Acids in the Hindgut of Rats Fed Processed Bean (*Phaseolus vulgaris*) Flours Varying in Distribution and Content of Indigestible Carbohydrates. Br. J. Nutr..

[69] Jan G., Belzacq A.S., Haouzi D., Rouault A., Métivier D., Kroemer G., Brenner C. (2002). Propionibacteria Induce Apoptosis of Colorectal Carcinoma Cells via Short-Chain Fatty Acids Acting on Mitochondria. Cell Death Differ..

[70] Nogal A., Valdes A.M., Menni C. (2021). The Role of Short-Chain Fatty Acids in the Interplay between Gut Microbiota and Diet in Cardio-Metabolic Health. Gut Microbes.

[71] Vanderhaeghen S., Lacroix C., Schwab C. (2015). Methanogen Communities in Stools of Humans of Different Age and Health Status and Co-Occurrence with Bacteria. FEMS Microbiol. Lett..

[72] Bereswill S., Fischer A., Plickert R., Haag L.M., Otto B., Kühl A.A., Dashti J.I., Zautner A.E., Muñoz M., Loddenkemper C. (2011). Novel Murine Infection Models Provide Deep Insights into the “Ménage à Trois” of Campylobacter Jejuni, Microbiota and Host Innate Immunity. PLoS ONE.

[73] Wang L., Zhang J., Guo Z., Kwok L., Ma C., Zhang W., Lv Q., Huang W., Zhang H. (2014). Effect of Oral Consumption of Probiotic Lactobacillus Planatarum P-8 on Fecal Microbiota, SIgA, SCFAs, and TBAs of Adults of Different Ages. Nutrition.

[74] Pérez-Burillo S., Pastoriza S., Gironés A., Avellaneda A., Pilar Francino M., Rufián-Henares J.A. (2020). Potential Probiotic Salami with Dietary Fiber Modulates Metabolism and Gut Microbiota in a Human Intervention Study. J. Funct. Foods.

[75] Van den Abbeele P., Gérard P., Rabot S., Bruneau A., El Aidy S., Derrien M., Kleerebezem M., Zoetendal E.G., Smidt H., Verstraete W. (2011). Arabinoxylans and Inulin Differentially Modulate the Mucosal and Luminal Gut Microbiota and Mucin-Degradation in Humanized Rats. Environ. Microbiol..

[76] Ríos-Covián D., Ruas-Madiedo P., Margolles A., Gueimonde M., De los Reyes-Gavilán C.G., Salazar N. (2016). Intestinal Short Chain Fatty Acids and Their Link with Diet and Human Health. Front. Microbiol..

[77] Del Mar Rigo-Adrover M., Franch À., Castell M., Pérez-Cano F.J. (2016). Preclinical Immunomodulation by the Probiotic Bifidobacterium Breve M-16V in Early Life. PLoS ONE.

[78] Morales-Ferré C., Azagra-Boronat I., Massot-Cladera M., Tims S., Knipping K., Garssen J., Knol J., Franch À., Castell M., Rodríguez-Lagunas M.J. (2021). Effects of a Postbiotic and Prebiotic Mixture on Suckling Rats’ Microbiota and Immunity. Nutrients.

[79] Rinne M., Kalliomaki M., Arvilommi H., Salminen S., Isolauri E. (2005). Effect of Probiotics and Breastfeeding on the Bifidobacterium and Lactobacillus/Enterococcus Microbiota and Humoral Immune Responses. J. Pediatr..

[80] der Balian G.P., Slack J., Clevinger B.L., Bazin H., Davie J.M. (1980). Subclass Restriction of Murine Antibodies. III. Antigens That Stimulate IgG3 in Mice Stimulate IgG2c in Rats. J. Exp. Med..

[81] Harmer N.J., Chahwan R. (2016). Isotype Switching: Mouse IgG3 Constant Region Drives Increased Affinity for Polysaccharide Antigens. Virulence.

[82] Wallace J.M., Milne J.S., Aitken R.P., Redmer D.A., Reynolds L.P., Luther J.S., Horgan G.W., Adam C.L. (2015). Undernutrition and Stage of Gestation Influence Fetal Adipose Tissue Gene Expression. J. Mol. Endocrinol..

[83] Zhang Y., Otomaru K., Oshima K., Goto Y., Oshima I., Muroya S., Sano M., Roh S., Gotoh T. (2022). Maternal Nutrition During Gestation Alters Histochemical Properties, and MRNA and MicroRNA Expression in Adipose Tissue of Wagyu Fetuses. Front. Endocrinol..

[84] Lukaszewski M.A., Eberlé D., Vieau D., Breton C. (2013). Nutritional Manipulations in the Perinatal Period Program Adipose Tissue in Offspring. Am. J. Physiol. Endocrinol. Metab..

