# Supplementary material for: Maternal Synbiotic Supplementation with B. breve M-16V and scGOS/lcFOS Shape Offspring Immune Development and Gut Microbiota at the End of Suckling
Source: Nutrients. 2024 Jun 15;16(12):1890. doi: 10.3390/nu16121890 (PMC11206815; doi:10.3390/nu16121890)
Supplement: Supplementary file 1 [file nutrients-16-01890-s001.zip › nutrients-3006893-supplementary.pdf]

Supplementary Materials

Supplementary Tables

Supplementary Table 1. Description of the specific TaqMan primers AB

Gene	Reference
Tlr2	Rn02133647_s1, I
Tlr3	Rn01488472_g1, I
Tlr4	Rn00569848_m1, I
Tlr5	Rn04219239_s1, I
Tlr7	Rn01771083_s1, I
Tlr9	Rn01640054_m1, I
Muc2	Rn01498206_m1, I
Muc3	Rn01481134_m1, I
Ocln	Rn00580064_m1, I
Cldn2	Rn02063575_s1, I
Cldn4	Rn01196224_s1, I
Tjp1	Rn02116071_s1, I, encoding for Zo1
IgA	331943, made to order
Prdm1	Rn03416161_m1, I, encoding for Blimp1
Fcgrt	Rn00566655_m1, I, encoding for FcRn
Gusb	Rn00566655_m1, I

I, inventoried

Supplementary Table 2. Growth-associated parameters and organ's size at the weaning day.

**Body size**	**REF**	**SYN**
Body lenta (cm)	18.67 ± 0.14	18.27 ± 0.22
Body/tail length ratio	1.57 ± 0.02	1.62 ± 0.02
BMI (g/cm^2)	0.35 ± 0.01	0.34 ± 0.01
Lee index ($g^{0.33}/cm \times 10^3$)	317.47 ± 5.76	314.97 ± 6.72

**Relative organ size**	**REF**	**SYN**
Spleen (%)	0.37 ± 0.02	0.35 ± 0.01
Thymus (%)	0.50 ± 0.01	0.52 ± 0.01
Kidney (%)	3.65 ± 0.11	3.63 ± 0.15
Heart (%)	0.62 ± 0.01	0.61 ± 0.02
Liver (%)	0.60 ± 0.02	0.60 ± 0.03
Salivary gland (%)	0.15 ± 0.01	0.15 ± 0.02
Stomach (%)	0.69 ± 0.05	0.69 ± 0.03
Caecum (%)	0.41 ± 0.02	0.30 ± 0.02*
Small intestine (%)	3.40 ± 0.08	4.12 ± 0.24*
Small intestine length (cm)	79.66 ± 1.87	88.79 ± 3.10*

Data are expressed as mean ± S.E.M. (n=11-16). Statistical differences: $^*p < 0.05$ vs REF.

Supplementary Table 3. Effect of maternal synbiotic supplementation on the spleen and MLN lymphocyte population at weaning.

	Spleen		MLN	
	REF	SYN	REF	SYN
B cells (CD45RA+)	35.63 ± 1.47	31.89 ± 1.06	21.43 ± 1.75	23.02 ± 3.18
% CD25+	12.08 ± 1.10	13.00 ± 1.45	5.25 ± 0.37	4.72 ± 0.71
T cells (TCRαβ/TCRγδ)	26.38 ± 1.50	30.33 ± 4.31	73.67 ± 2.03	71.47 ± 2.89
TCRαβ+ NK-	28.06 ± 3.61	28.19 ± 2.75	69.96 ± 1.99	68.33 ± 3.02
% CD8+	69.94 ± 0.91	65.95 ± 1.14*	75.48 ± 0.48	76.35 ± 0.65
TCRγδ+	2.72 ± 0.38	3.53 ± 0.30	3.71 ± 0.33	3.14 ± 0.46
% CD8+	2.27 ± 0.12	2.65 ± 0.27	2.13 ± 0.12	1.92 ± 0.20
CD4+ CD8-	25.27 ± 2.97	29.20 ± 2.58	52.24 ±2.11	46.51 ± 4.49
% CD25+	6.19 ± 0.59	5.39 ± 0.84	11.47 ± 0.57	13.30 ± 2.28
CD8+ CD4-	12.4 ± 0.61	13.01 ± 1.06	17.77 ± 0.69	16.59 ± 1.51
% CD25+	6.2 ± 1.24	6.93 ± 2.70	7.99 ± 0.60	8.41 ± 1.57
CD4+ CD8+	2.86 ± 0.22	4.01 ± 0.50*	2.26 ± 0.24	2.75 ± 0.49
% CD25+	6.20 ± 1.34	4.40 ± 1.08	41.39 ± 3.32	44.43 ± 7.99
NK (TCRαβ- NK+)	2.97 ± 0.34	3.74 ± 0.46	1.22 ± 0.13	1.75 ± 0.23*
% CD8+	21.13 ± 2.16	22.62 ± 2.15	11.99 ± 1.66	10.68 ± 1.85
NKT (TCRαβ+ NK+)	3.06 ± 0.13	3.30 ± 0.35	1.95 ± 0.12	2.20 ± 0.12
% CD8+	72.64 ± 3.01	78.65 ± 10.6	54.64 ± 1.36	46.00 ± 2.95*

Data are expressed as mean ± S.E.M. Statistical differences: *$p < 0.05$ vs REF (n=11-16).

Supplementary Table 4. Haematological variables at day 21 of pups' life.

Haematological variable	REF	SYN
Leucocytes (x10^9 /L)	2.95 ± 0.59	4.36 ± 1.01*
Lymphocytes (%)	69.81 ± 1.79	69.20 ± 2.50
Monocytes (%)	5.39 ± 0.58	5.76 ± 0.74
Granulocytes (%)	24.80 ± 1.50	25.04 ± 1.92
Lymphocytes (x10^9 /L)	1.58 ± 0.10	2.94 ± 0.71$^{\#}$
Monocytes (x10^9 /L)	0.13 ± 0.03	0.23 ± 0.09
Granulocytes (x10^9 /L)	0.88 ± 0.23	1.20 ± 0.28
Erythrocytes (x10^{12} /L)	4.25 ± 0.08	4.10 ± 0.32
HGB (g/L)	77.93 ± 1.49	151.82 ± 86.52
HCT (%)	23.11 ± 0.58	22.28 ± 1.75
VCM (fL)	54.49 ± 0.62	54.64 ± 0.87
MCH (pg)	18.31 ± 0.22	18.42 ± 0.28

Data are expressed as mean ± S.E.M (n=11-16). Statistical differences: $^{*}p < 0.05$ vs REF; $\#p < 0.1$ vs REF. HGB, Hemoglobin; HCT, Hematocrit; MCV, Mean Cell Volume; MCH, Mean Cell Hemoglobin.

Supplementary Figures

Supplementary Figure 1.

a) b)

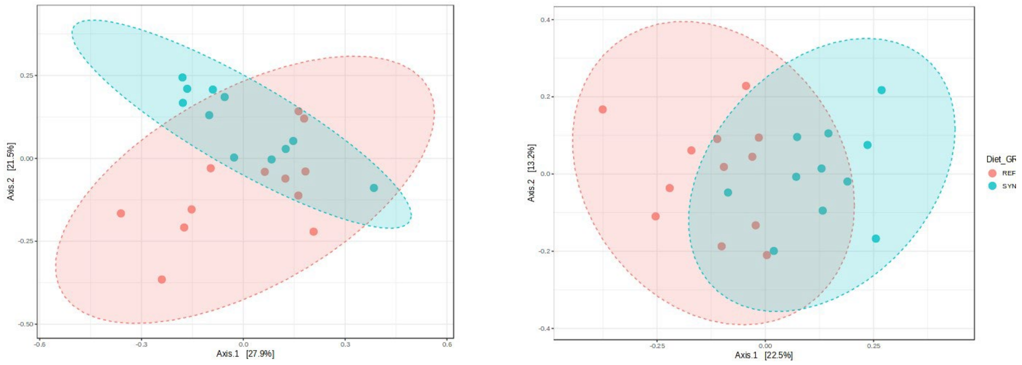

Supplementary Figure 1. Analysis of non-parametric multidimensional scaling (NMDS) for the microbiota profiles based on the Bray-Curtis distance in (a) IC and (b) CC. Each point in NMDS represents an animal by ANOSIM test. Statistical differences: *$p < 0.05$ vs REF (n=11-16).
